# Peripheral blood mononuclear cell gene expression and cytokine profiling in patients with intermittent claudication who exhibit exercise induced acute renal injury

**DOI:** 10.1371/journal.pone.0265393

**Published:** 2022-03-17

**Authors:** Pasha Normahani, Joseph J. Boyle, Luke Cave, Paul Brookes, Kevin J. Woollard, Usman Jaffer

**Affiliations:** 1 Imperial Vascular Unit, Imperial College Healthcare NHS Trust, London, United Kingdom; 2 Department of Surgery and Cancer, Imperial college London, London, United Kingdom; 3 National Heart and Ling Institute, Imperial College London, London, United Kingdom; 4 Department of Pathology, Royal Brompton and Harefield hospitals, London, United Kingdom; 5 Department of Immunology and Inflammation, Imperial College London, London, United Kingdom; Ludwig-Maximilians-Universitat Munchen, GERMANY

## Abstract

**Background:**

Intermittent claudication (IC) is a common manifestation of peripheral arterial disease. Some patients with IC experience a rise in Urinary N-acetyl-β-D-Glucosaminidase (NAG)/ Creatinine (Cr) ratio, a marker of renal injury, following exercise. In this study, we aim to investigate whether peripheral blood mononuclear cells (PBMC) from patients with IC who exhibit a rise in urinary NAG/ Cr ratio following exercise exhibit differential IL-10/ IL-12 ratio and gene expression compared to those who do not have a rise in NAG/ Cr ratio.

**Methods:**

We conducted a single center observational cohort study of patients diagnosed with IC. Blood and urine samples were collected at rest and following a standardised treadmill exercise protocol. For comparative analysis patients were separated into those with any rise in NAG/Cr ratio (Group 1) and those with no rise in NAG/Cr ratio (Group 2) post exercise. Isolated PBMC from pre- and post-exercise blood samples were analysed using flow cytometry. PBMC were also cultured for 20 hours to perform further analysis of IL-10 and IL-12 cytokine levels. RNA-sequencing analysis was performed to identify differentially expressed genes between the groups.

**Results:**

20 patients were recruited (Group 1, n = 8; Group 2, n = 12). We observed a significantly higher IL-10/IL-12 ratio in cell supernatant from participants in Group 1, as compared to Group 2, on exercise at 20 hours incubation; 47.24 (IQR 9.70–65.83) vs 6.13 (4.88–12.24), p = 0.04. 328 genes were significantly differentially expressed between Group 1 and 2. The modulated genes had signatures encompassing hypoxia, metabolic adaptation to starvation, inflammatory activation, renal protection, and oxidative stress.

**Discussion:**

Our results suggest that some patients with IC have an altered immune status making them ‘vulnerable’ to systemic inflammation and renal injury following exercise. We have identified a panel of genes which are differentially expressed in this group of patients.

## 1. Introduction

Peripheral arterial disease (PAD) is characterised by narrowing or occlusion of peripheral arteries and is estimated to effect over 230 million people worldwide [1]. Intermittent claudication (IC), which is defined as exertional pain in the lower extremity that is relieved by rest, is a common manifestation of PAD. Patients with IC have a three- to six- fold increased mortality from cardiovascular events compared with age matched controls [2]. This elevated risk may be related to the systemic endothelial dysfunction and inflammation associated with PAD which can accelerate existing cardiovascular disease [3–6]. This may be linked to reduced vessel wall shear stress and repeated ischemia-reperfusion injury [7] which has been observed to improve following revascularisation [8]. In patients with IC, exercise results in tissue ischaemia with local accumulation of anaerobic metabolites and oxygen free radicals. This episodic ischaemia results in these metabolites circulating and resulting in a transient systemic inflammatory response and end organ injury [9–11].

We have previously shown that a subgroup of patients with IC experience a rise in Urinary N-acetyl-β-D-Glucosaminidase (NAG)/ Creatinine (Cr) ratio, a sensitive and specific marker of renal injury, following exercise [11]. This group also demonstrated a lower level of baseline oxidative stress, higher post exercise increase in oxidative stress and macro-vascular endothelial function [11]. We proposed that this group may represent a phenotypically distinct subset of patients with IC who are more susceptible to acute kidney injury, and possibly other end organ injury, following exercise.

Human peripheral blood mononuclear cells (PBMC) are composed of lymphocytes (T cells, B cells, and NK cells), monocytes, and dendritic cells. They are an important component of innate immunity and play a key role in inflammation and the oxidative stress response. Differences in PBMC gene expression have been demonstrated between patients with and without PAD [12]. Furthermore, both IL-10 (Interleukin 10) and IL-12 (Interleukin 12) have important roles in the regulation of the innate immune system [13] and have been associated with the inflammatory state of atherosclerosis observed in PAD [14]. In this study, we aim to investigate whether PBMC, from patients diagnosed with IC, who exhibit a rise in urinary NAG/ Cr ratio following exercise exhibit differential IL-10/ IL-12 and gene expression compared to individuals who do not have a rise in NAG/ Cr ratio. This may help identify useful biomarkers for patients with IC who are susceptible to exercise induced end organ injury, and also potentially provide useful insights into underlying molecular mechanisms.

## 2. Materials and methods

We conducted a single center observational cohort study. The study was approved by the United Kingdom National Research Ethics Committee (REC 12/WA/0196). All recruited patients provided written informed consent.

### 2.1 Patient recruitment

Potential participants were identified from the prospectively managed arterial database of patients with IC undergoing routine clinical follow up at Hammersmith Hospital, London. All patients with evidence of haemodynamically significant lower limb PAD on duplex ultrasonography and documented current symptoms of intermittent claudication were invited to participate. Patients with signs or symptoms suggestive of chronic limb threatening ischaemia (CLTI) were not eligible for inclusion. Patients were excluded in the presence of concomitant disease severe enough to preclude treadmill exercise (such as musculoskeletal pain, heart disease or lung disease). Dialysis-dependent renal failure patients were also excluded. Patient demographics, comorbidities and medications as well as ankle-brachial pressure indices were recorded.

### 2.2 Treadmill exercise protocol

A standard testing protocol was followed: Ambulant patients were asked to empty their bladder before a 90-minute rest period (to avoid interference from the exercise of walking to the vascular laboratory). Following rest, urine and blood samples were collected and temporarily stored on ice. The participant was then asked to walk briskly on a treadmill (10 per cent incline; 4 miles/ hour) until they could not tolerate their typical claudication pain. Further blood and urine samples were taken and temporarily stored on ice.

Protease inhibitor cocktail (5% weight/volume; Sigma-Aldrich, Gillingham, UK) was added to urine samples which were stored at -70°C and analysed collectively. Urinary NAG and Cr levels were measured using ELISA. The NAG/Cr ratio was calculated to normalise for differences in urine output rate.

### 2.3 Peripheral blood mononuclear cell (PBMC) separation and immunofluorescence flow cytometry

A modified Boyum’s density gradient separation method was used to isolate PBMC. Briefly, 20 ml of blood collected in an EDTA tube was layered onto an equal volume of Lymphoprep (Axis-Shield UK, Cambridgeshire, UK) and centrifuged at 2800 rpm for 20 minutes. The ‘buffy coat’ was removed and diluted to 20 ml with Gibco RPMI 1640 medium (Life technologies ltd, Manchester, UK). This was further centrifuged at 2200 rpm, supernatant tipped off and cell pellet re-suspend and allowed to stand for 5 minutes in 5 ml of 0.85% Ammonium chloride to cause red cell lysis. The cell suspension was again diluted to 20 ml with RPMI and centrifuged at 1500 rpm for 5 minutes. Again, supernatant was tipped off and cells re-suspend in 5 ml of RPMI with 5% foetal calf serum, 1% glutamine, and 1% penicillin/ streptomycin (Sigma-Aldrich; Dorset, England). Cell concentration was determined using a haemocytometer and 5000 cell events were recorded for each sample.

For flow cytometric analysis 200μl of cell suspension was pipetted into 5 wells of V bottomed 96 well plates, which were centrifuged at 1500 rpm for 5 minutes. Supernatant was discarded and cell pellet re-suspended in 100μl of phosphate buffered saline (PBS) and again centrifuged at 1500 rpm for 5 minutes. The cell pellets were re-suspended in 40μl PBS and either 10μl of PBS or a two-colour direct immunofluorescence reagent in a 1:5 dilution (BD: Simultest™ CD3/CD16 + CD56; Simultest™ LeucoGATE™ (CD45/ CD14); Oncomark CD14/ CD64; Becton Dickson, Oxford, UK) was added and incubated at 4°C for 30 minutes. Samples were transferred to test tubes and 600μl PBS added. The percentage of fluorescent cells of each leukocyte population was quantified using a Beckman Coulter FC500 instrument (Beckman-Coulter, High Wycombe, UK).

### 2.4 Cytokine assay from PBMC

0.5ml of the PBMC suspension, containing 1 X 10^6^ cells, was seeded in 92mm polystyrene cell culture dishes (Nunclon; Fisher Scientific, Loughborough, UK) and incubation was performed after Signorelli et al. [15] for 20 hours at 37°C in a moist chamber containing 5% CO_2_. 50μl of supernatant was collected after 1-, 6- and 20-hours incubation and stored at -70°C for ELISA. A custom quantitative multiplex ELISA protocol modified for low sample volume was used to analyse IL-12p70 (dynamic range 3000 to 5.35 pg/ml) and IL-10 (dynamic range 2680 to 4.78 pg/ml) from supernatant samples (Q-plex; Quansys Biosciences, Logan, Utah, USA).

### 2.5 Peripheral blood mononuclear cell (PBMC) RNA-sequencing

**Fig 1:**
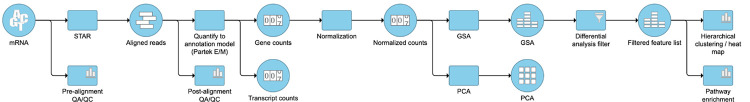
Schematic of workflow within Partek. Blue boxes, processes as indicated. Blue circles, data types as indicated.

Isolated PBMC were lysed in RNA-lysis buffer, RNA purified and analysed by RNA-Sequencing (**Fig 1)**. After RNA-sequencing was performed, read files were quality checked, processed and analysed using Partek^®^ Flow^®^ software (Version 7, Partek Inc., St. Louis, MO, USA). Read quality tests showed that both the average base quality score per position and the average quality score per read was above 30 (i.e., 99.9% base call accuracy), indicating that files were of good quality. Alignment was performed using the STAR aligner tool [16]. Post-alignment quality tests demonstrated that at least 75% of reads were aligned to the genome per sample and that samples had an average read depth of a covered region of at least 30. The aligned reads were quantified to the Partek^®^ E/M annotation model producing number of counts per gene. Gene counts were normalised to read depth per sample, using the counts per million (CPM) method. The statistical comparison test, gene-specific analysis, was performed between the two test groups resulting in a fold change value, p-value and FDR-value per gene.

Significantly regulated genes were analysed using hierarchical cluster analysis and principal component analysis, Gene Ontology analysis by PANTHER GO-Slim (Version 14) [17–19], network analysis by mapping onto the STRING database (Version 11) [20] and graphing in Cytoscape (Version 3.8) [21], and transcription factor binding site analysis, using a summation of rank scores from OPOSSUM with JASPAR core matrices, OPOSSUM with JASPAR position weight matrices, X2K with each of ENCODE+ChEA, ENCODE, ChEA2015, JASPAR + TRANSFAC, ChEA2016, ARCHS4, ENRICHR, ChEA3 and PASTAA.

### 2.6 Statistical analysis

Data were analysed using Microsoft Excel (2010 edition) and SPSS (version 20). Difference between groups was tested using the Mann Whitney U test (two-tailed) for numerical values and Chi-squared for categorical data. p values of ≤0.05 were considered statistically significant.

## 3. Results

Twenty patients participated in this study. Patient demographics are presented in **Table 1**. Overall, across all participants median urinary NAG/Cr rose from a pre-exercise level of 8.94 (IQR, 6.73 to 14.27) to a post exercise level of 12.90 (IQR 9.07 to 17.72); p = 0.0003. Patients were separated into two groups for comparative analysis; those with any rise in NAG/Cr ratio post exercise (**Group 1**; n = 8) and those with no rise in NAG/Cr ratio (**Group 2**; n = 12).

**Table 1:** Summary of patient demographics.

	Total	Group 1	Group 2	P value
Total number of participants	20	8	12	
Age (median, IQR)	72 (66–80)	68 (63–73)	75 (66–80)	0.25
Gender = male (n, %)	11 (55%)	5 (63%)	6 (50%)	0.54
Average ABPI (median, IQR)	0.8 (0.7–0.9)	0.71 (0.65–0.83)	0.83 (0.72–0.89)	0.61
Maximum walking distance (metres) (median, IQR)	265 (155–450)	395 (283–716)	195 (140–303)	0.05
History of ischaemic heart disease	6 (30%)	2 (25%)	4 (33%)	0.96
History of Diabetes	6 (30%)	2 (25%)	4 (33%)	0.82
Statin therapy = Yes	12 (60%)	4 (50%)	8 (67%)	0.78
Antiplatelet therapy = Yes	12 (60%)	5 (63%)	7 (58%)	1

### 3.1 PBMC composition

**Fig 2:**
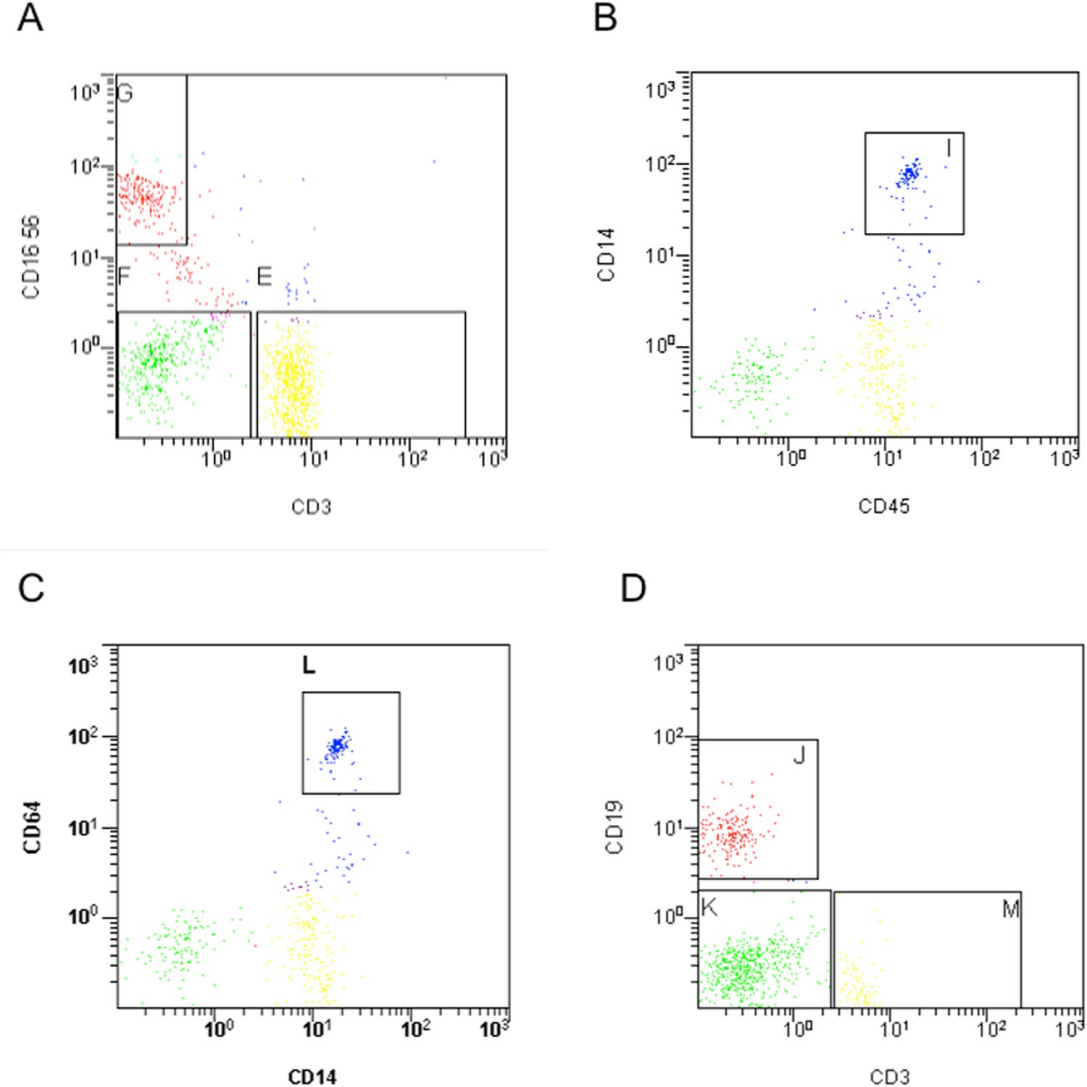
Representative plots from flow cytometric analysis. **A)** CD3+/16-/56- cells (gate E), CD16+/56+/3- cells (gate G); **B)** CD45+/14+ cells (gate I); **C)** CD14+/64+ cells (gate L); **D)** CD19+/3- cells (gate J). We gated for the whole population from the Lymphoprep isolation; forward and side scatter plots is available in the **S1 Fig**.

**Fig 3:**
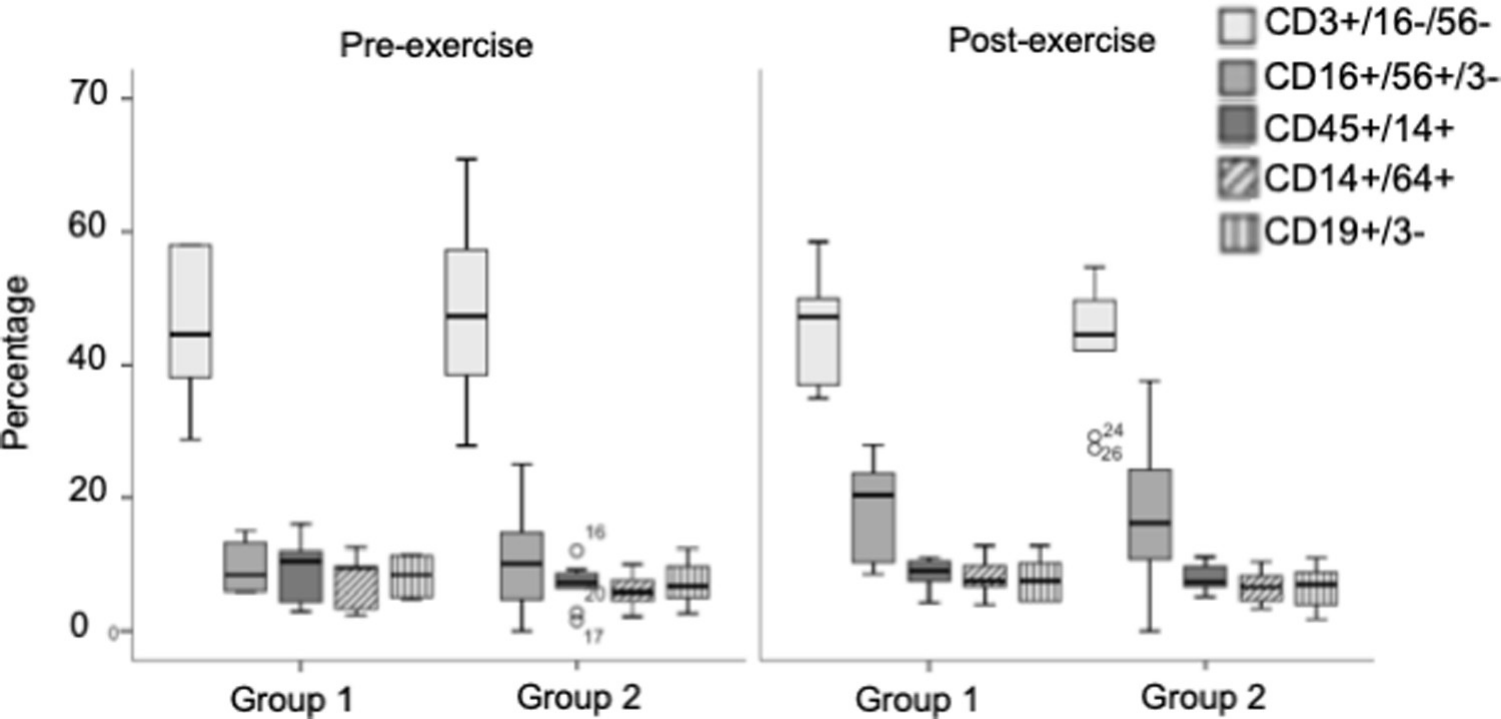
Percentage composition of PBMC in claudicants pre- and post-exercise separated by those who experiences a rise in urinary NAG/ Creatinine ratio (Group 1) vs. no rise on exercise (Group 2).

The pre-exercise total mononuclear cell counts were similar between participants in Group 1 (3.825 x 10^6^; 2.575 x10^6^ to 4.925 x10^6^) and Group 2 (2.975 x10^6^; 2.500 x10^6^ to 4.950 x10^6^) (cells/ ml, median; IQR). The post-exercise cell counts were also similar between participants in Group 1 (3.400 x10^6^; 1.475 x10^6^ to 9.338 x10^6^) and Group 2 (4.800 x10^6^; 3.575 x10^6^ to 6.388 x10^6^) (cells/ ml, median; IQR). Typical gates employed for flow cytometric analysis are demonstrated (**Fig 2)**. Flow cytometric analysis demonstrated that the PBMC percentage composition was similar between participants in Group 1 and Group 2 (**Fig 3**).

### 3.2 PBMC supernatant cytokine profile

**Fig 4:**
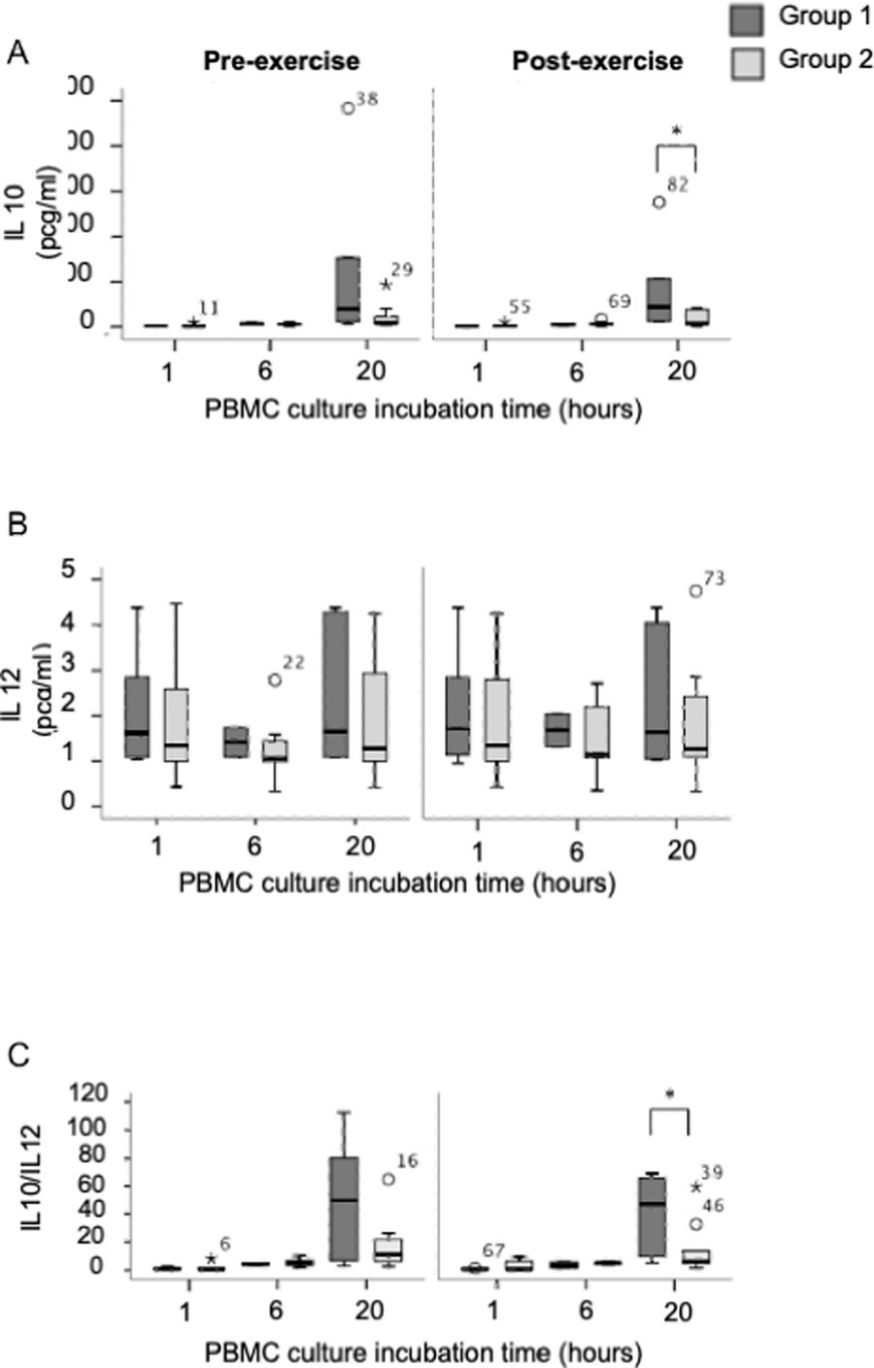
**A)** IL-10 (pcg/ ml), * p = 0.045 **B)** IL-12 (pcg/ ml), **C)** IL-10/IL-12 ratio from cell medium supernatant of PBMC cultured from patients with (Group 1) and without (Group 2) rise in urinary NAG/Creatinine ratio following exercise, * p = 0.04. Statistical comparisons made using Mann Whitney U test.

Supernatant from PBMC from pre-exercise blood samples did not demonstrate any statistically significant difference in IL-10 or IL-12 levels or IL-10/IL-12 ratio between participants in Group 1 and Group 2. However, post-exercise sample supernatant contained higher levels of IL-10 in Group 1 as compared to Group 2 at 20 hours following incubation (44.36, 15.11 to 96.27 versus 7.27, 5.24 to 38.74; pcg/ml/10^6^ cells; median, IQR; p = 0.045; **Fig 4A**). IL-12 levels did not exhibit any difference between Group 1 and 2: 1.64, 1.17 to 3.47 versus 1.27, 1.09 to 2.43 (pcg/ml/10^6^ cells; median, IQR; p = 0.724; **Fig 4B**).

Interestingly, there was a significantly higher IL-10/IL-12 ratio in cell supernatant from participants in Group 1, as compared to Group 2, on exercise at 20 hours incubation: 47.24, 9.70 to 65.83 versus 6.13, 4.88 to 12.24 (median, IQR; p = 0.04; **Fig 4C**). There was no difference in IL-10/IL-12 ratio from supernatant of PBMC taken from pre-exercise blood at one, six or 20 hours of incubation between participants in Group 1 and 2. Similarly, there were no differences seen in supernatant taken from post exercise samples at one- or six-hours following incubation.

### 3.3 PBMC RNA-sequencing

**Fig 5:**
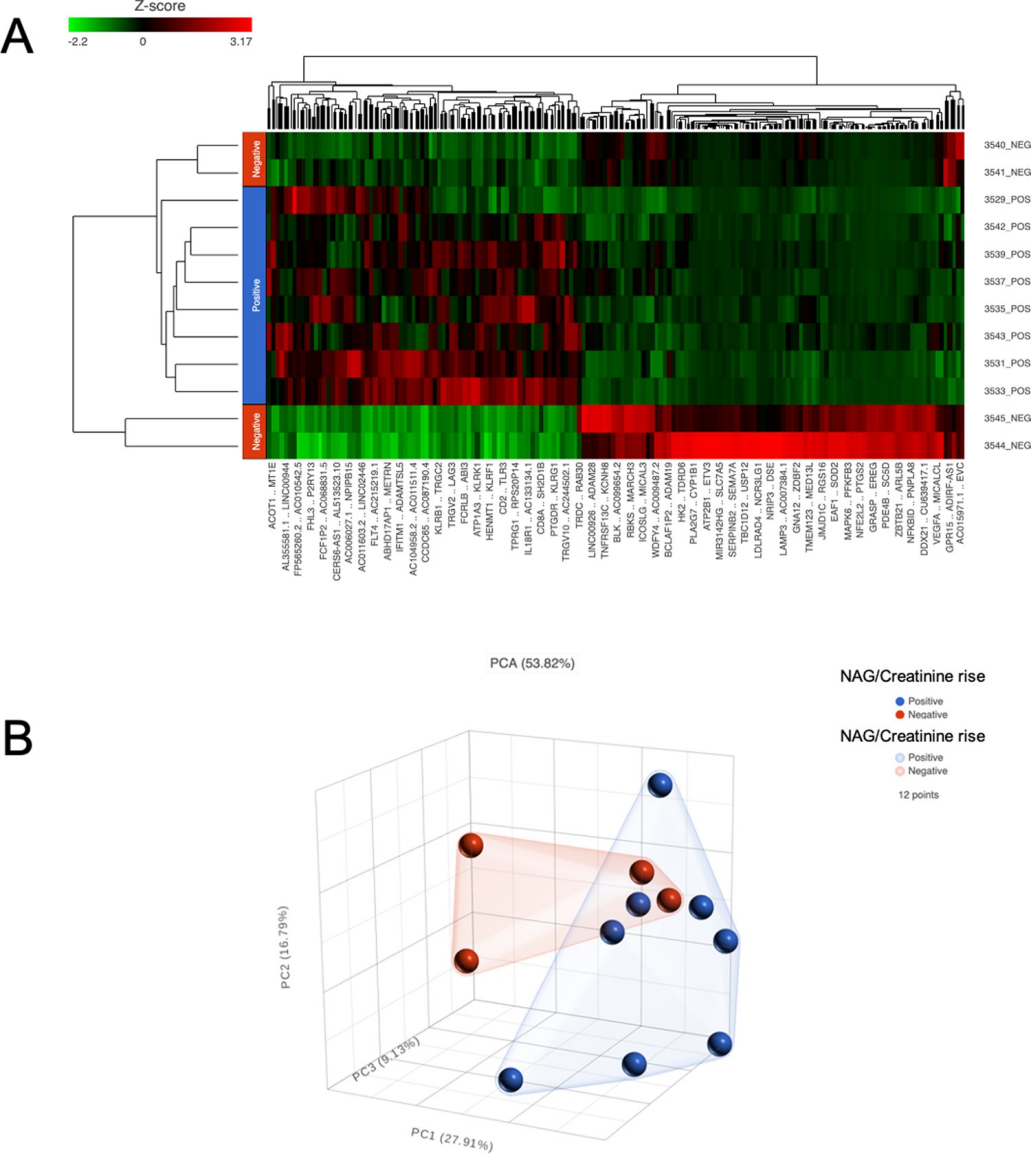
**A)** hierarchical cluster analysis. Upregulated genes (red) and downregulated genes (green) as indicated. There is a clear separation between the groups with (POS) and without (NEG) urinary NAG/Creatinine rise following exercise. The group with a rise urinary NAG/ Creatinine has a cluster of upregulated genes to the right-hand side of the diagram. **B)** principal component analysis. There is a clear separation between the groups with (blue, POS) and without (red, NEG) urinary NAG/ Creatinine rise following exercise. The groupings are shown with the superimposed polygons.

**Fig 6:**
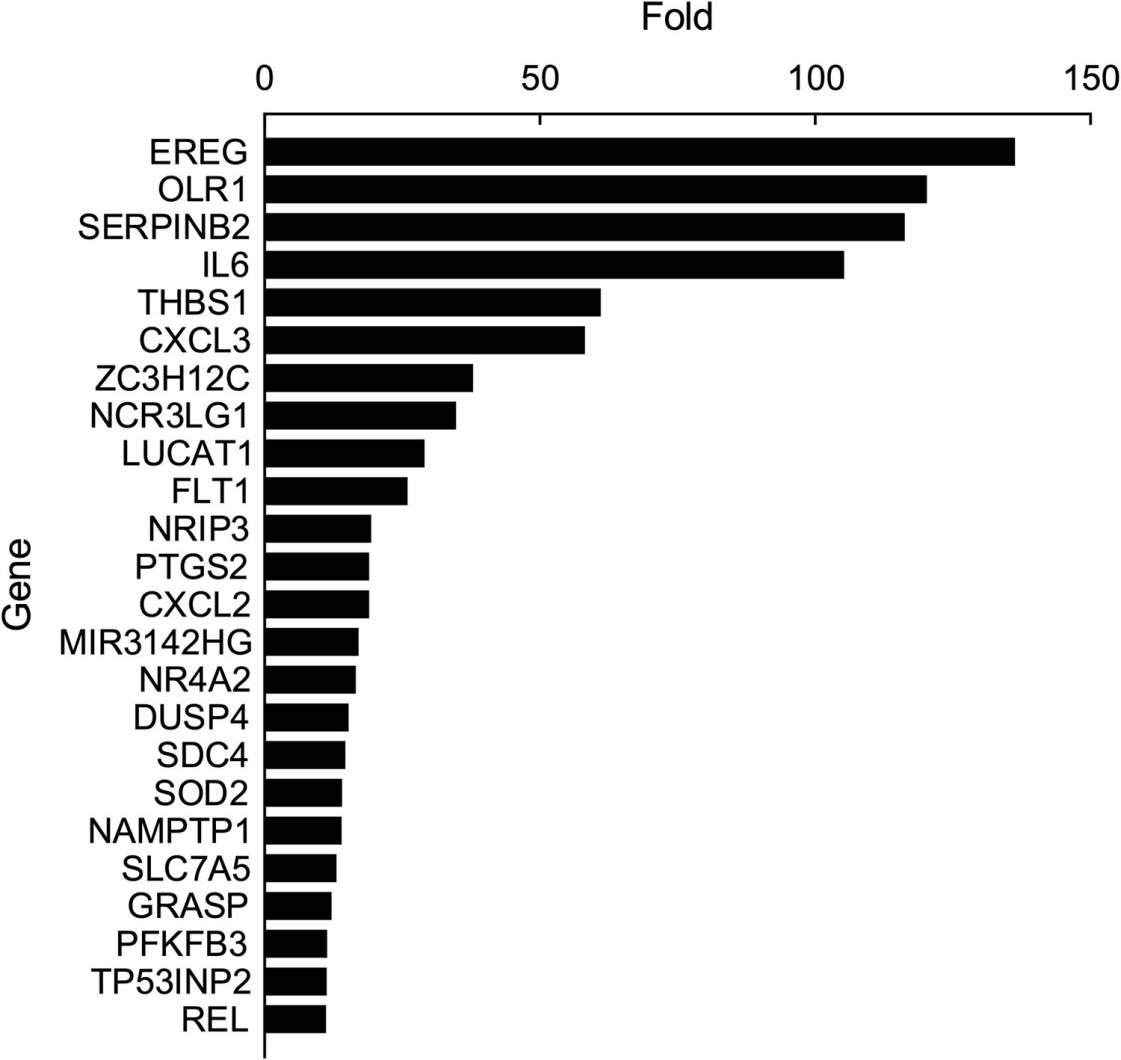
List of top 24 genes by fold up regulation.

**Fig 7:**
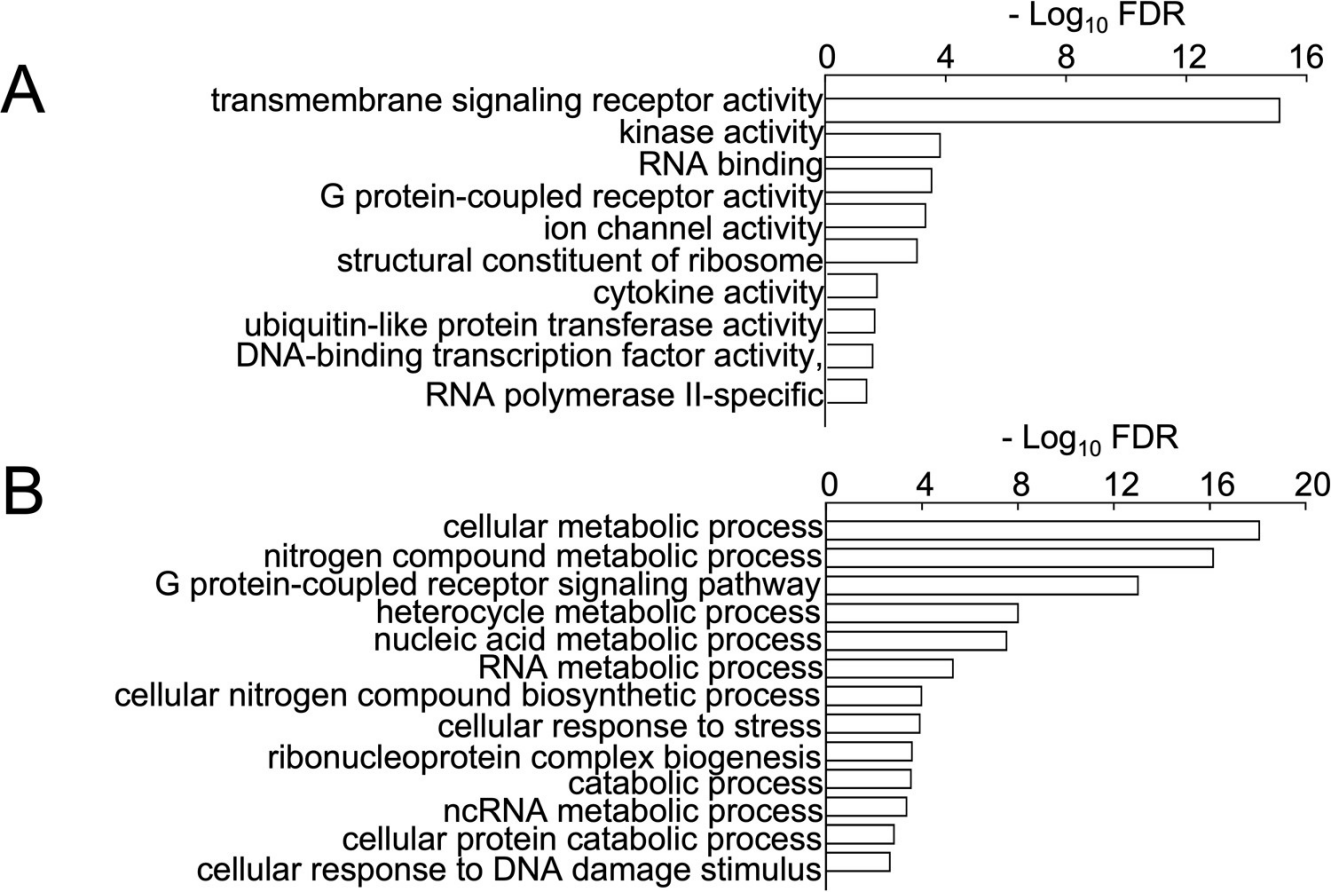
**A)** Y-axis, important GO categories of Molecular Function; X-axis, probability expressed as negative log of the false discovery rate (FDR). **B)** Y-axis, important GO categories of Cellular Function; X-axis, expressed as negative log of the false discovery rate (FDR).

328 genes were significantly differentially expressed between Group 1 and 2 (**Fig 5A**). Significantly differentially expressed genes (**Fig 6**) were assessed by Gene Ontology (GO) analysis. Significant GO categories for Molecular Function are shown (**Fig 7A)**. For space and clarity, they are reduced to the distinct and meaningful categories in the list. Signal transduction was a notable molecular function. Specific signal transduction activities included kinases, GPCR (G-Protein Coupled Receptor) activity, ion channels, cytokine activity, ubiquitinylation, transcription factor activity. Significant GO categories for Cellular Processes are shown (**Fig 7B**). These included a set of metabolic categories: Nitrogen compound metabolism; Heterocycle metabolic processes; Nucleic acid metabolic processes; RNA metabolic processes; Nitrogen compound biosynthesis; Ribonucleoprotein biosynthesis; Catabolic processes; ncRNA metabolic processes. Additional notable GO categories were cellular responses to stress, and to DNA damage.

#### 3.3.1 Network analysis

**Fig 8:**
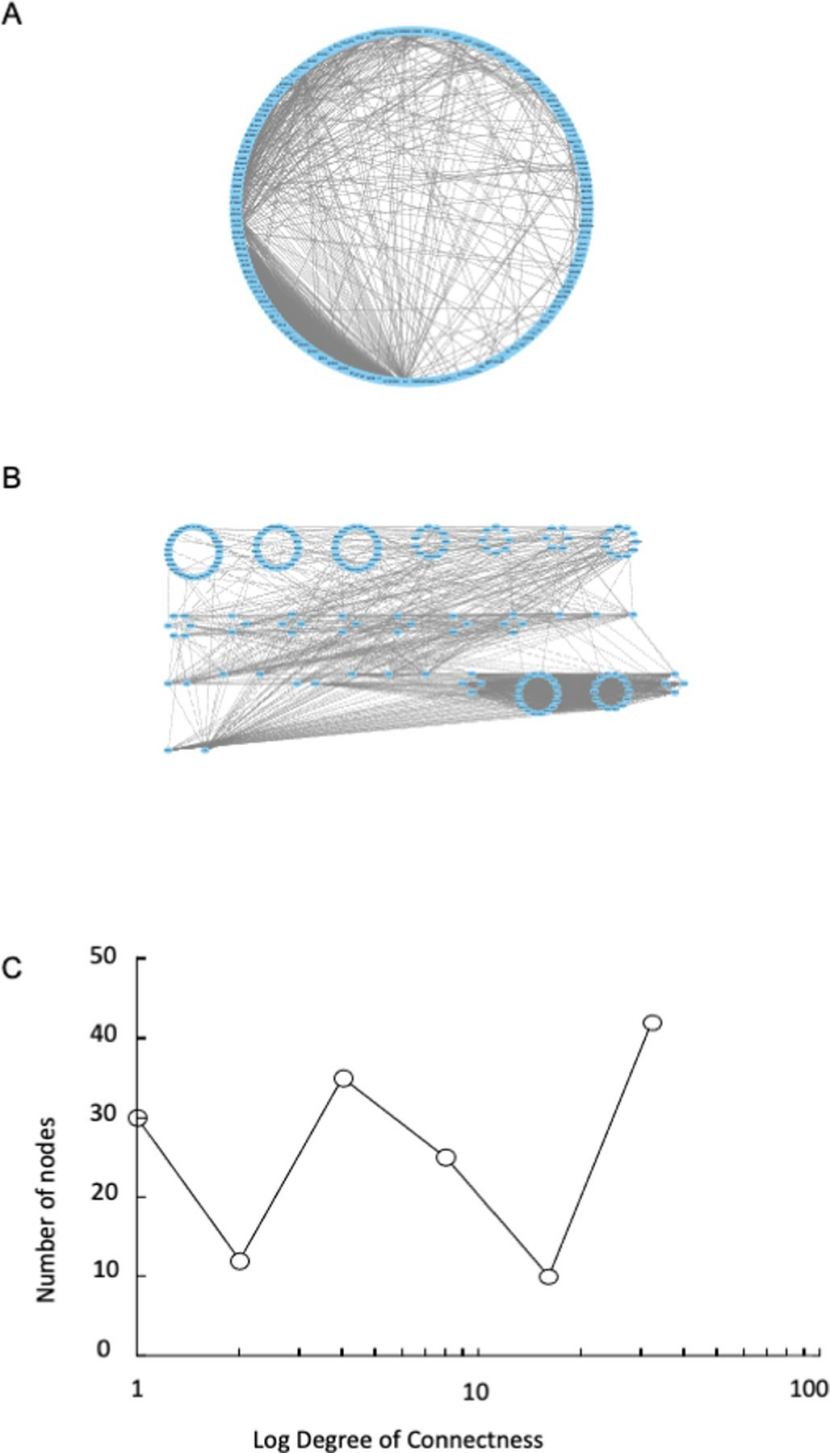
**A)** overview of gene network. Blue, nodes in the network. Grey lines, known connections between nodes. Some nodes are very highly connected. **B)** alternate overview of gene network, redrawn to maximally display connections and the most highly connected nodes. Blue, nodes in the network. Grey lines, known connections between nodes. Some nodes are very highly connected. **C)** graph of node connections. X-axis, number of connections per node; Y-axis, number of nodes.

Network analysis was assessed to help to identify the most connected genes in a process, which are more likely to be important regulators. As only a single time point was available, the differentially expressed genes were mapped onto the database of protein-protein interactions. The most highly connected genes (**Fig 8A, 8B**) included *RPS2 (Ribosomal Protein S2)*, *IL6 (Interleukin 6)*, *PTGS2 (Prostaglandin Synthase 2)*, *CCL5 (C-C Chemokine Ligand 5)*, *RPP25 (Ribonuclease P and MRP subunit P25)*, *SOD2 (Superoxide Dismutase 2)*, *CCR7 (C-C Motif Chemokine Receptor 7)*, *VEGF (Vascular Endothelial Growth Factor)*, and a family of ribosomal proteins. The two most highly connected nodes were *IL6* and *RPS2*. These data were consistent with the GO data pointing to transmembrane signal transduction, cytokine signalling, kinase activity and ribonucleoprotein and ribosomal biogenesis. The number of connexions for each node (degree of connectedness) was then output from Cytoscape and collated into ‘bins’ to smooth out random variations in connections per node. These bins were on a logarithmic scale because node degree and number of nodes typically forms a straight line on that graph (the power law relationship). Then connectedness was graphed against the number of nodes (**Fig 8C**). The graph was highly non-linear, with 2 unexpected peaks, one in the mid-range and one comprising highly connected nodes.

**Fig 9:**
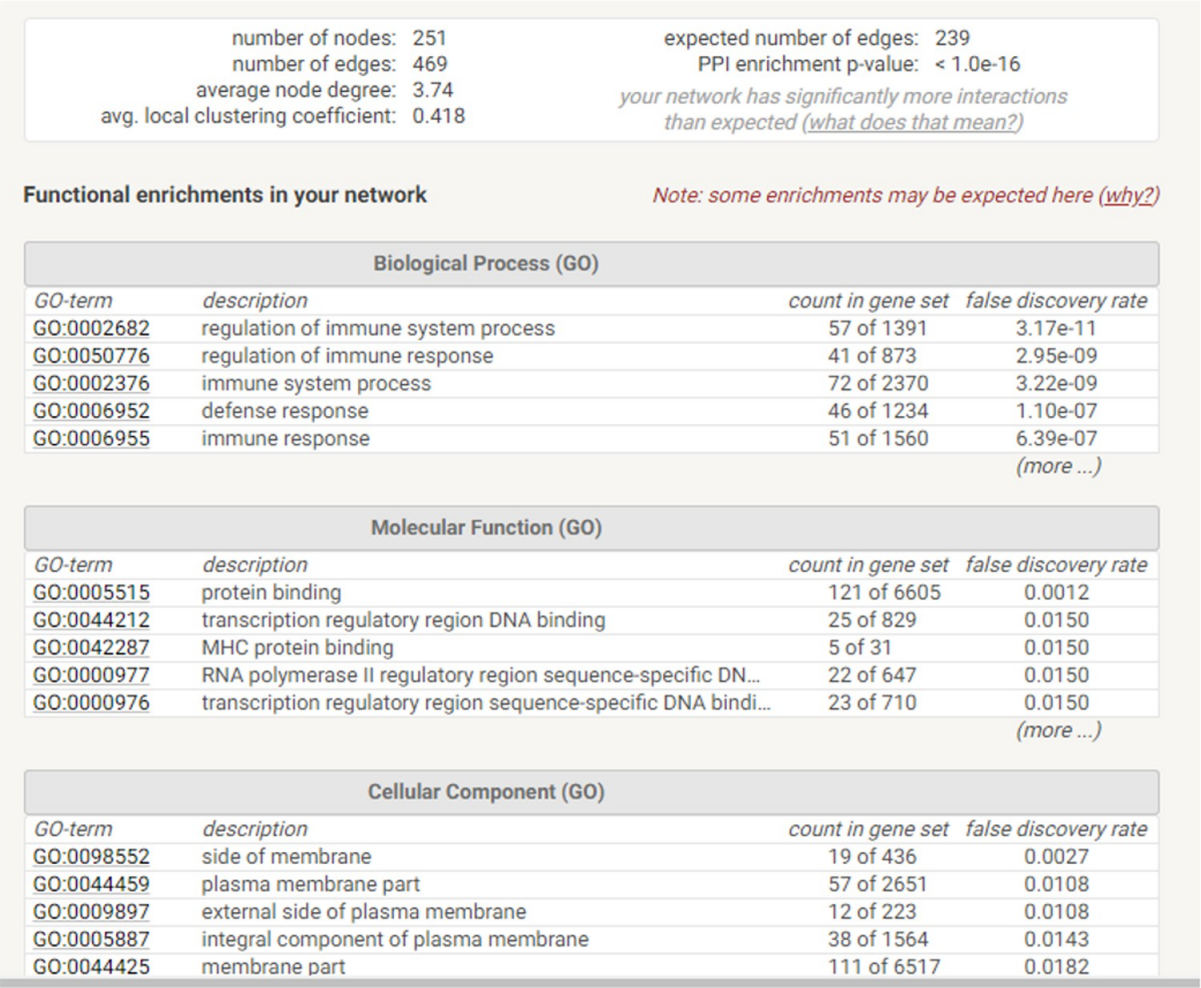
STRING network summary. The summary data are shown at the top and summarise the graphs in Fig 7.

The STRING database also output a summary of the network (**Fig 9**). This indicated over-representation for regulation of immune system and transcription factor binding in Biological Process (**Fig 9**). Molecular Function showed enrichment for Transcription Factors (**Fig 9**).

#### 3.3.2 Transcription factor binding site analysis

**Fig 10:**
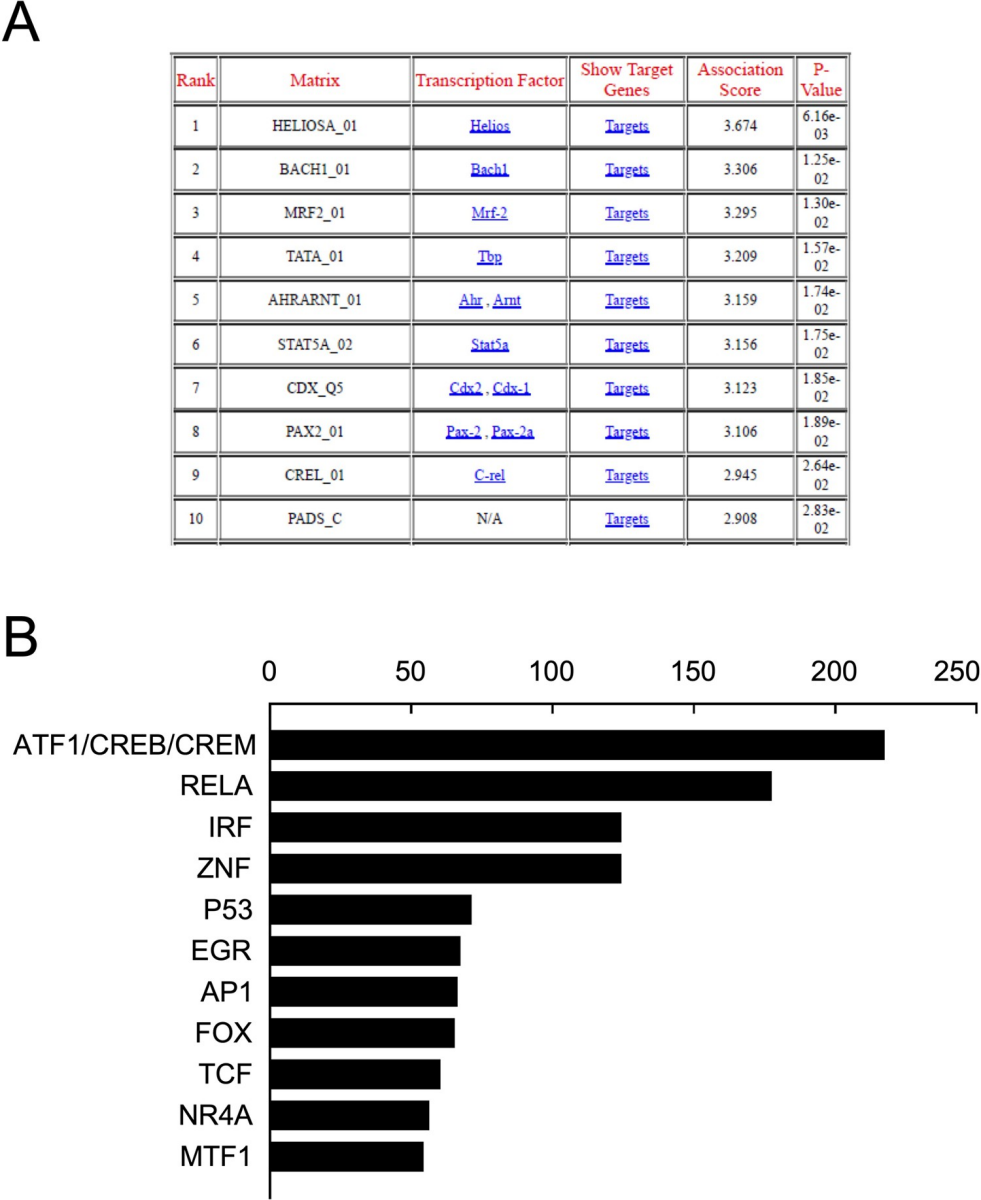
**A)** list of predicted Transcription Factor binding sites (PASTAA) ranked by P-value (generated using the Max Planck Institute for Molecular Genetics Database), **B)** X-axis, score calculated as an aggregate of rankings of common transcription factor binding sites prediction programs (OPPOSUM using JASPAR, JASPAR CORE, X2K using databases ENCODE, ChEA2015, JASPAR and TRANSFAC, ChEA2016, archs4, ENRICHR, ChEA3, PASTAA).

We then assessed by transcription factor binding site (TFBS) analysis. Different TFBS analysis programs may yield different results. Therefore, multiple analysis programs were used. Output for PASTAA is shown (**Fig 10A**). This showed over-representation for binding sites for ATF, CREB (which is related to ATF), CREL (NFkB), SP1, EGR3, SRF, KROX, STAT, AHRARNT. Output TFBS scores were ranked for each and combined **(Fig 10B)**. Top scoring TFs across 11 programs and databases indicated that the most highly scored TFBS representing subfamilies of transcription factors were CREB1 (which is related to ATF1 and CREM), NFkB (CREL), IRF, ZNF, P53, EGR, AP1, FOX, TCF, NR4A and MTF1.

## 4. Discussion

This study has detected increased levels of the anti-inflammatory IL-10 and significantly higher IL-10/ IL-12 ratio in the PBMC supernatant of patients with IC who exhibited evidence of acute renal injury following exercise. We also conducted gene expression profiling of sampled PBMC which demonstrated a complex systemic inflammatory process in this group of patients. Our results suggest that some patients with IC may have an altered immune status which contributes to systemic inflammation and acute renal injury following exercise.

Our previous profiling of this cohort of patients suggested that those who exhibit exercise induced NAG/Cr rise also have a greater rise in oxidative stress (hydrogen peroxide; H_2_O_2_) and a greater increase in endothelial reactivity (flow mediated dilation of the brachial artery) following exercise as compared to those with no NAG/Cr rise [11]. Interestingly, this group also demonstrated a longer average walking distances on the treadmill (395m vs 195m; p = 0.05) despite comparable disease severity, as measured by the ankle-brachial pressure index (0.71 vs 0.83; p = 0.61), as compared to those with no NAG/Cr rise in this previous study [11].

Consistent with the literature, there was a small but non-significant increase in CD16+ cells post exercise, likely to be CD16+ monocytes [22, 23]. To avoid over-reliance on one type of bioinformatic analysis, several different approaches were taken, allowing themes common to the majority to emerge. Gene Ontology analysis pointed to an important molecular role for signal transduction. The signature of a cytokine pathway in this analysis could relate to the other signatures for IL6 as an important node, and STAT sites in the clusters of regulated genes. The signature indicating use of GPCRs could also be related to PTGS2 (which generates both pro-inflammatory and anti-inflammatory eicosanoids) and to CRE sites, as eicosanoids signal in part via GPCRs coupled to adenylate cyclase and cyclic-AMP.

Activation of the central pro-inflammatory NF-κB pathway involves multiple cell surface receptors, kinases, and ubiquitinylation of the inhibitory component IκBα. Thus, the ubiquitinylation, kinase and transmembrane signalling GO hits are likely to represent involvement of NF-κB.

IL-6 was prominent on some of the RNA-seq analysis, particularly in the network analysis as a highly connected gene. IL-6 may now be specifically inhibited by neutralising antibodies, that are licensed for inflammatory disease [24]. As IL-6 elevates so-called acute phase markers. Serum CRP (C-Reactive Protein) would be an interesting follow-up and has been linked to long-term risk of atherosclerotic cardiovascular disease [25].

The GO for biological process was also insightful. As a form of ischemia-reperfusion, claudication could be expected to activate cellular processes involved in cell energy deprivation. It is interesting that related signals could be detected in peripheral blood, indicating systemic activation. The PBMC sampled had GO signals for catabolism including protein catabolism. In addition, the GO signatures suggested a commitment to RNA synthesis. This would be in keeping with the other signatures for transcription factor activation and gene regulatory activity and is likely to be involved in a systemic response to claudication. It could reflect gene transcription for pro-inflammatory activity, pro-repair activity or metabolic adaptation.

Taken together, the modulated genes had signatures encompassing hypoxia, metabolic adaptation to starvation, inflammatory activation, renal protection, and oxidative stress. Some of these pathways were pointed to by a combination of induced transcription factor and induced target genes, such as NFκB driven inflammation (namely C-REL (p65 NFκB) and target genes IL6, CXCL3 (CXC-Motif Chemokine Ligand 3), NFκB, PTGS2, CXCL2 (CXC-Motif Chemokine Ligand 2), DUSP4 (Dual Specificity Phosphatase 4) and SOD2). Similarly, induction of hypoxia-inducible factor (HIF), and some of its target genes (VEGFA, HIF1A, SEMA7A (Semaphorin 7A), SLC2A3 (Solute Carrier Family 2 member A3), HK2 (Hexokinase 3), PFK (Phosphofructokinase)) pointed to a role for responses to hypoxia, as expected in ischemia [26]. The catabolic response, suggested involvement of the ischemia-activated signalling kinase AMPK (5’ Adenosine Monophosphate Activated Protein Kinase) as a driver as it drives catabolism [27]. Some of the genes and predicted transcription factors are related to oxidative stress, notably SOD2, BACH1, NFE2L2, MAFG. This ties in with our previous description of elevated oxidative stress in these patients. Of interest was the novel protective gene NAMPT (Nicotinamide Phosphoribosyl Transferase), which has been shown to be protective in renal ischemia [28]. CREB target genes were also prominent and statistically significant (NR4A2, PURB, RBKS, RPP25, PTPRK, DTX4, QPRT, EAF1, TRAF4, STYK1, TGIF1, JMJD1C, PDE4B, BANK1), consistent with a role for cyclic-AMP activators such as eicosanoids derived from PTGS2 (COX2).

A potential limitation of this work is that enriched PBMC’s may include a proportion of other contaminating cells. In future studies, flow cytometric analysis may be beneficial to quantify this. However, all samples were treated similarly and so any potential contamination would not be expected to confound our findings.

## 5. Conclusions

Our results suggest that some patients with IC have an altered immune status making them ‘vulnerable’ to systemic inflammation and end organ injury following exercise. We have identified a panel of genes which are differentially expressed in this vulnerable group of patients and may be potentially useful for clinical screening. Further validation is necessary to evaluate the utility of this gene panel as a prognostic biomarker for patients with intermittent claudication.

## Supporting information

S1 Fig Forward scatter vs side scatter plot for cytometric analysis. (DOCX)
