# Supplementary figures and images for: Peripheral blood mononuclear cell gene expression and cytokine profiling in patients with intermittent claudication who exhibit exercise induced acute renal injury
Source: PLoS One. 2022 Mar 17;17(3):e0265393. doi: 10.1371/journal.pone.0265393 (PMC8929566; doi:10.1371/journal.pone.0265393)

**S1 Fig.** Forward scatter vs side scatter plot


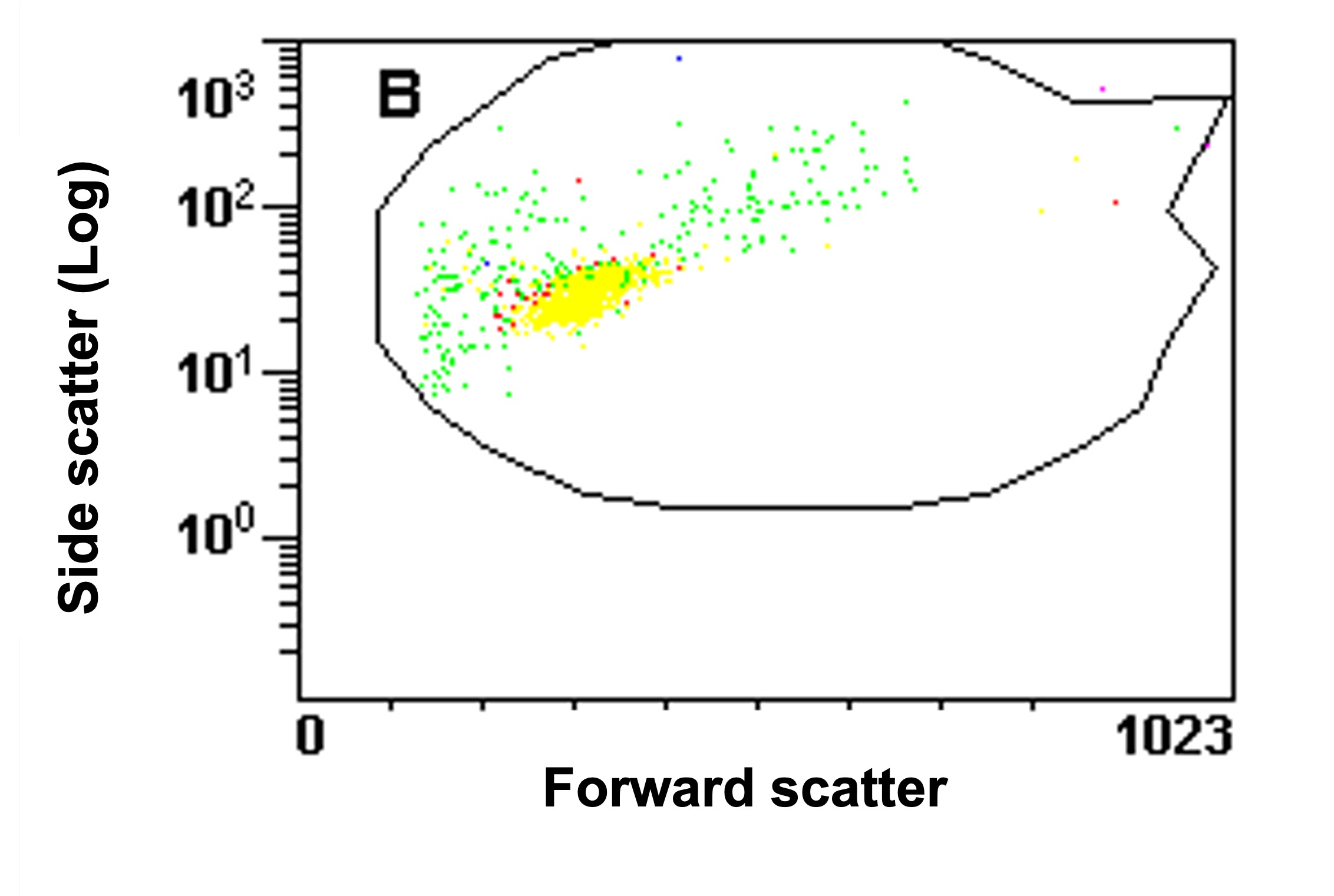

Supplement: S1 Fig — (DOCX) [file pone.0265393.s001.docx]
